# Supplementary material for: Nickel price forecasting based onempirical mode decomposition and deep learning model with expansion mechanism
Source: PLoS One. 2026 Mar 24;21(3):e0341559. doi: 10.1371/journal.pone.0341559 (PMC13012476; doi:10.1371/journal.pone.0341559)
Supplement: S1 Appendix — This appendix presents the pseudo-code, metric alignment, EEMD and dilated LSTM settings, leakage safeguards, Diebold–Mariano test, and SHAP aggregation details. (PDF) [file pone.0341559.s001.pdf]

S1 Appendix. Implementation details for the walk-forward evaluation. This appendix presents the pseudo-code, metric alignment, EEMD and dilated LSTM settings, leakage safeguards, Diebold–Mariano test, and SHAP aggregation details.

A.1 Rolling-origin procedure (pseudo-code).

Let $\mathcal{T}_{\text{train}}$, $\mathcal{T}_{\text{val}}$, $\mathcal{T}_{\text{test}}$ be contiguous time blocks; let $\{t_k\}_{k=1}^{K}$ be re-estimation dates inside $\mathcal{T}_{\text{test}}$ at ~20-trading-day intervals.

1.For each $k = 1, \ldots, K$:

a. Define window $W_k = [\text{start}, t_k]$ (anchored expanding).

b. Fit preprocessing on W_k (training portion only); transform inputs/targets for W_k and the upcoming evaluation span.

c. Train the model (hyper-parameters fixed from validation).

d. Forecast for each evaluation day $t \in (t_k, t_{k+1}]$: output $\hat{y}_{t+h}$ for $h = 1, \ldots, 7$.

2.Concatenate forecasts across k to form continuous test-set predictions per horizon h.

3.Evaluate metrics per h; run horizon-wise DM tests vs. benchmarks.

A.2 Metrics and alignment.

For each horizon h, the evaluation set contains only dates where both y_{t+h} and $\hat{y}_{t+h}$ exist (no look-ahead). We report MAPE/MAE/RMSE/R per horizon and average across horizons when summarizing. Confidence bands are obtained via the 10-seed dispersion; significance is assessed via DM.

A.3 EEMD and dilation settings.

1.EEMD: noise amplitude uniformly sampled within $[0.1,0.4]\sigma$ across ensembles; 100 realizations averaged to form each IMF component.

2.Dilated LSTM: three-stage dilation (1–2–4) to expand the receptive field while preserving parameter efficiency; hidden width and dropout as specified in Methods, held fixed across refits.

A.4 Leakage safeguards.

All transformations (scaling, EEMD noise level reference) use statistics from the training part of W_k only. Hyper-parameters are not re-tuned on the test period; re-estimation retrains weights but does not search hyper-parameters.

A.5 Statistical testing details.

DM tests use the same loss as the metric under test (e.g., absolute error for MAE), with Newey–West lag equal to horizon h to account for overlapping forecast errors. We report two-sided p-values.

A.6 SHAP aggregation.

SHAP is computed on the test forecasts; per-feature attributions are averaged over seeds and summarized by channel groups (IMFs vs. residual; nickel vs. exogenous metals). We report top-k contributors and cumulative shares to characterize frequency-band dominance.
