# Supplementary material for: Nickel price forecasting based onempirical mode decomposition and deep learning model with expansion mechanism
Source: PLoS One. 2026 Mar 24;21(3):e0341559. doi: 10.1371/journal.pone.0341559 (PMC13012476; doi:10.1371/journal.pone.0341559)
Supplement: Attachment — Submitted filename: Response to reviewers.doc [file pone.0341559.s003.doc]

Dear Editor and Reviewers,

Thank you for your professional and thoughtful comments on our manuscript. We have strengthened the paper along three axes: rigorous tests of forecast‐accuracy differences, control of uncertainty arising from random initialization, and an economics-grounded interpretation of the SHAP results. Below we provide a cohesive account of what we changed and the new evidence supporting our conclusions.

*Reviewer comment:* *1) The manuscript reports only point estimates (MAPE, RMSE, R) for in- and out-of-sample performance but provides no statistical significance tests of error differences across models. I recommend performing Diebold-Mariano or Clark-West pairwise tests between EEMD-DilatedLSTM and the second-best models (e.g., Dilated-GRU, PatchTST) at every horizon and adding p-values to Table 4-1 to confirm that the superiority is not driven by random noise.*

**Response:** Regarding statistical significance, at each forecast horizon we identify the strongest competitor by the lowest MAPE and conduct a pairwise comparison with EEMD–DilatedLSTM. Concretely, the benchmark is NHITS at h=1, Dilated-GRU at h=2 through h=6, and GRU at h=7. We apply the Diebold–Mariano test with Newey–West heteroskedasticity–autocorrelation adjustment to accommodate the serial correlation induced by overlapping multi-step forecast errors. The loss measure is the absolute percentage error to align the test with the MAPE criterion used for ranking. Results are summarized in Table 4-2: all horizons yield p<0.05. This demonstrates that the improvements delivered by EEMD–DilatedLSTM over the strongest competitor at each horizon are statistically reliable rather than artifacts of random noise. We have also clarified the full testing workflow and parameter choices in the Methods section so that this verification is reproducible.

*Reviewer comment: The authors argue that “a single training run already contains multi-step rolling errors, hence no repetitions are needed”; however, this only reflects the distribution of sample errors and does not reveal variance due to parameter initialization under different random seeds. I advise running at least ten independent trainings and reporting mean ± standard deviation (or 95% confidence intervals) of MAPE/R to rule out “lucky initialization”.*

**Response:** To address the legitimate concern about “lucky initialization,” we retrained every model at every horizon ten times under independent random seeds, while holding constant the data splits, scaler fitting on the training set only, the optimizer, and early-stopping criteria. Table 4-2 reports the mean and standard deviation of MAPE, RMSE, and R by horizon; upon request we can also provide 95% confidence intervals derived from these repeated runs. The dispersion across seeds is very small—MAPE standard deviations are a small fraction of their means, and the standard deviations of R remain minimal—showing that both performance levels and model rankings are stable under repetition. In combination with the significance tests, this multi-seed evidence provides a second, independent pillar: the differences are statistically significant and they are reproducible across independent trainings.

*Reviewer comment:* *3) Although SHAP identifies IMF_8/9 as the most influential features, the text interprets them generically as “long-term trends”. A narrative linking these 2–4-year low-frequency components to nickel-market fundamentals (mine expansion cycles, EV policy shifts, inventory structures) with citations to industry studies would strengthen economic interpretability rather than purely statistical interpretability.*

**Response:** On the economic interpretation of the SHAP results, Section 4.3 now moves beyond the generic label of “long-term trend.” We show that IMF_8 and IMF_9, which dominate the marginal contributions for medium-to-long horizons, align with verifiable mechanisms in the nickel value chain that operate with structural, multi-year lags. On the supply side, the path from investment to steady output—especially in NPI and HPAL—proceeds through financing, construction, commissioning, and ramp-up, creating slow capacity cycles that transmit to prices via expectations, spot imbalances, and re-equilibration; these dynamics are captured in the terminal IMFs as low-frequency undulations. On the demand side, periodic reevaluation of fiscal incentives, rolling updates to carbon and fuel-economy standards, the redesign of subsidy and access windows, and cathode-mix shifts between NMC/NCA and LFP produce multi-year recalibration rhythms that leave a low-frequency imprint on nickel intermediates and refined products. Inventories and the term structure add another slow channel: restocking–destocking cycles co-move with contango and backwardation, yielding inventory–spot patterns that evolve over several years. To quantify this alignment, we estimate dominant frequencies via the Hilbert spectrum and report pseudo-periods; empirically, IMF_8 and IMF_9 concentrate around two to four years, consistent with the three slow processes above. This chain of evidence indicates that our medium-to-long-horizon gains arise not from abstract smoothing but from EEMD’s extraction of industry rhythms, which the Dilated-LSTM then exploits through its enlarged temporal receptive field to provide stable trajectory constraints. By contrast, short-horizon accuracy relies more on mid- and high-frequency IMFs and lagged cross-metal signals, which better capture near-term co-movements and the rapid propagation of news and inventory shocks. The statistical and economic narratives therefore converge and help explain why every horizon in Table 4-2 attains significance.

All concrete revisions and additional experiments are clearly marked in the tracked-changes version and mapped to the relevant sections, tables, and figures. We have responded point-by-point with a sincere and rigorous effort: ten independent trainings per model with mean and standard deviation reporting in Table 4-2, pairwise Diebold–Mariano tests with Newey–West adjustment against the strongest competitor at each horizon with p-values reported in Table 4-2, and an expanded SHAP analysis that aligns dominant low-frequency IMFs with supply ramp-ups, policy and technology recalibration, and inventory–term-structure cycles. We also clarified data splitting and walk-forward evaluation, the exclusive use of lagged exogenous variables to prevent leakage, and the recording of seeds and hyperparameters for full reproducibility.

In sum, the revisions materially enhance the paper’s rigor and persuasiveness. The significance tests establish that the performance differences are genuine; the ten-run summary statistics and confidence quantification show that the results are reproducible and robust; and the economics-aware SHAP analysis demonstrates that the gains stem from identifiable industry rhythms rather than incidental curve-fitting. We believe these changes make the methodology and interpretation more complete and provide actionable insight for hedging tenor selection, inventory timing, and policy-window assessment. We appreciate your careful review and stand ready to implement any additional refinements you may recommend.
