# Supplementary material for: Nickel price forecasting based onempirical mode decomposition and deep learning model with expansion mechanism
Source: PLoS One. 2026 Mar 24;21(3):e0341559. doi: 10.1371/journal.pone.0341559 (PMC13012476; doi:10.1371/journal.pone.0341559)
Supplement: Attachment — Submitted filename: response to reviewers.docx [file pone.0341559.s004.docx]

Response to Reviewer #3

We appreciate your careful reading and the ambitious extensions you suggest. Our manuscript is positioned as a price-level forecasting study for nickel futures over short-to-medium horizons (h = 1–7), built around a hybrid EEMD–Dilated LSTM that addresses nonstationarity, nonlinearity, and long memory in daily commodity prices. Within this scope, we (i) evaluate against strong baselines over multiple horizons, (ii) run ablations to isolate the contribution of decomposition and dilation as well as multivariate co-traded metals, and (iii) provide economic interpretability via SHAP. The data (2015–2024, nickel + seven metals) already span multiple market regimes. The additional items you propose—crisis-window tests, macro/policy factor blocks with feature selection, parameter-surface mapping, long-horizon design, volatility forecasting, and trading backtests—together would substantially alter the problem statement and evaluation protocol. Below we indicate text clarifications we add now and extensions we move to Limitations & Future Work to preserve a coherent contribution and reasonable length.

1) Robustness under extreme market conditions

Commen: Evaluate forecasting performance separately over extreme periods (COVID-19 2020; Russia–Ukraine 2022), compare metrics normal vs. stressed, and discuss shock transmission.

Response: Our evaluation period (2015–2024) already traverses both tranquil and stressed regimes; the walk-forward test therefore reflects model behavior through these episodes. We agree that a pre-registered event-window analysis would be informative, but doing it credibly entails careful window definition, counterfactual timing, and guarding against ex-post bias.

Manuscript change: We now state explicitly in Results that the test period spans COVID-19 and the 2022 conflict and note observed stability at h = 1–7. We also add two sentences in Discussion clarifying why we did not split the test by events in this paper and commit a dedicated crisis-window module to Limitations & Future Work (window design, metrics, and an outline of nickel’s strategic-material transmission channels).

2) Inclusion of macroeconomic/policy variables with selection (e.g., rates, inflation, DXY, CRB, EV subsidies; stepwise/LASSO)

Comment: Add macro/policy drivers and select optimal subsets to improve interpretability and accuracy.

Response: We intentionally restricted exogenous features to co-traded metals to avoid daily release-calendar misalignment and leakage; a macro/policy block requires calendar-aware lags, revision handling, and stability checks to be credible at the daily frequency. We support this direction but see it as a scope expansion best handled as a follow-up.

Manuscript change: Methods now make this design choice explicit (why metals-only, how we control leakage). Discussion adds a short scope statement and moves the macro-augmented, LASSO-screened variant to Limitations & Future Work (with explicit notes on calendar alignment and anti-leakage safeguards).

3) Systematic parameter-sensitivity analysis (EEMD noise amplitude; dilation schedule)

Comment: Provide grid-search surfaces and stability intervals.

Response: We currently specify EEMD noise 0.1–0.4·σ with 100 ensembles and a 1–2–4 dilation that balances receptive field and efficiency; a full surface mapping across decomposition and dilation hyper-parameters would notably expand the paper and is better suited to a methodological companion.

Manuscript change: We add textual rationales for the chosen ranges in Methods and describe how a future parameter-surface study would be staged (axes, metrics, early-stopping policy, cross-seed aggregation). A full grid/BO exploration is recorded in Limitations & Future Work.

4) Medium/long-term horizons (≈20 and ≈60 trading days) and institutional benchmarks

Comment: Extend to 1- and 3-month horizons and compare attenuation vs. institutional forecasts.

Response: We agree on practical value; however, those horizons typically require different re-estimation cadence and loss calibration, risking protocol mixing with our current h = 1–7 design. To preserve coherence, we keep this paper focused on short-to-medium horizons that are most actionable for procurement and near-term hedging, and stage a dedicated horizon-extension study.

Manuscript change: Discussion now acknowledges the need and moves the 20/60-step evaluation (with benchmark comparisons and degradation profiling) to Limitations & Future Work.

5) Volatility forecasting vs. GARCH/SV with realized volatility

Comment: Add a volatility module, use RV targets, and compare MAE/MSE (or QLIKE) to GARCH/SV.

Response: Volatility forecasting is a distinct target with different data (preferably intraday RV) and different loss functions and baselines. We frame the present paper purposely as a price-level forecasting study to keep the evaluation clean and reproducible on daily data.

Manuscript change: Introduction and Discussion now contain explicit scope sentences distinguishing price-level from volatility targets and placing a volatility module with RV + GARCH/SV into Limitations & Future Work.

6) Backtesting of trading strategies (returns, Sharpe, max drawdown; costs/margins)

Comment: Convert forecasts to long-short strategies with realistic frictions and report performance.

Response: Strategy PnL depends heavily on microstructure assumptions (signal filtering, slippage and fee model, leverage/margin policy, drawdown controls) that can overshadow pure forecast quality. To avoid conflating econometrics with deployment choices, we defer a risk-controlled backtest framework to a separate, application-oriented paper.

Manuscript change: Discussion now explicitly delineates this boundary and records a standardized backtest plan (cost scenarios, turnover governance, retraining triggers, and risk limits) in Limitations & Future Work.

7) Transparency of rolling prediction (window size, refit cadence, reproducibility)

Comment: Specify the rolling-window design and analyze the impact of different settings.

Response: We agree that this should be fully explicit.

Manuscript change (implemented now): We added a new “Evaluation Protocol (walk-forward, multi-horizon)” subsection in Methods and a detailed Appendix. These text additions formalize (i) the chronological train/validation/test split, (ii) anchored expanding windows with monthly re-estimation in the test phase (≈ every 20 trading days) and full retraining at each anchor, (iii) strict leakage control (scaling on the training window only), (iv) multi-horizon forecasting for h = 1–7 on the held-out sequence, (v) 10 independent seeds with mean ± sd reporting, (vi) Diebold–Mariano tests with Newey–West adjustment per horizon against each benchmark, and (vii) SHAP computed on test forecasts and aggregated across seeds. These are textual clarifications of the pipeline already used; they do not alter results.

Across earlier rounds we materially improved the manuscript without changing its scope: (a) statistical robustness via multi-seed training and horizon-wise DM tests; (b) methodological clarity with explicit EEMD and dilation settings and unified preprocessing; and (c) economic interpretability showing low-frequency IMFs and specific co-traded metals (notably copper) as leading contributors at medium horizons. These upgrades increase credibility and readability while keeping the paper’s focus on price-level forecasting over h = 1–7 with a decomposition-plus-dilation architecture.

Limitations & Future Work

This study targets price-level forecasting over short-to-medium horizons (h = 1–7) using market-observable co-traded metals as exogenous inputs. Several valuable extensions are left for future work. First, we will design pre-registered extreme-event windows (e.g., COVID-19 in 2020; Russia–Ukraine in 2022) to compare performance in normal vs. stressed markets. Second, we will introduce macro and policy variables (policy rates, DXY, CRB, EV-subsidy intensity) with release-calendar alignment and LASSO/stepwise selection to enhance interpretability while guarding against leakage. Third, we will conduct parameter-surface studies for EEMD noise amplitude and dilation schedules. Fourth, we will extend to 1- and 3-month horizons and benchmark horizon-specific degradation against institutional reports. Fifth, we will add a volatility-forecasting module with realized-volatility targets and GARCH/SV baselines. Finally, we will translate forecasts into risk-controlled backtests (transaction costs, margin constraints, Sharpe, and maximum drawdown). The present paper’s multi-horizon comparisons, ablations, and SHAP diagnostics on 2015–2024 data provide a focused foundation for these application-oriented extensions.
